# Management of male premature ejaculation: from past to future

**DOI:** 10.3389/fendo.2025.1718109

**Published:** 2025-12-03

**Authors:** Jiaqing Chang, Weiwei Zhao, Lili Ma, Juan Zhao, Qiming Li, Xueyang Wang, Haichao Ju, Xinping Wang, Xing Xiping

**Affiliations:** 1 College of Integrative Chinese and Western Medicine, Gansu University of Chinese Medicine, Lanzhou, Gansu, China; 2 Imaging Center, Lanzhou First People’s Hospital, Lanzhou, Gansu, China; 3 Department of Andrology/Urology, Affiliated Hospital of Gansu University of Chinese Medicine, Lanzhou, Gansu, China

**Keywords:** premature ejaculation, pharmacological treatment, psychological and behavioral therapies, physical therapy, surgical treatment, traditional chinese medicine treatment

## Abstract

Premature ejaculation (PE) is a common disease of the male reproductive system, which seriously affects the quality of life of patients and their partners. Currently, PE is regarded as a biopsychosocial disease with complex etiologies and diverse treatment methods. Oral selective serotonin reuptake inhibitors (SSRIs) are the first-line treatment for PE, with advantages such as high safety, rapid onset of action, and non-invasiveness. However, topical anesthetics, behavioral and psychological therapies, device-assisted treatments, and traditional Chinese medicine (TCM) can also serve as alternative therapies for patients intolerant to SSRIs. With the rapid development of technology, some new methods—such as low-intensity extracorporeal shock wave therapy (Li-ESWT) and transcutaneous electrical nerve stimulation (TENS)—can even improve PE through mechanisms like regulating nerve conduction and improving local microcirculation. These are all important directions for the future treatment of male PE. In this mini-review, we will elaborate on these therapeutic approaches.

## Introduction

PE is defined by the presence of difficulty in controlling ejaculation, very short intravaginal ejaculatory latency time, associated with personal or partner distress, for a duration greater than six months (1). PE is a common male issue across all age groups, significantly impacting the psychological state and quality of life of both patients and their partners. It is reported that 20%-30% of men worldwide are affected by varying degrees of PE (2). Due to racial and regional differences, as well as varying definitions of PE, existing epidemiological data show considerable disparities. Guidelines from the ISSM and EAU indicate overall prevalence rates of 19.8% in Turkey and 25.8% in China, respectively (3, 4). Studies have shown that the occurrence of PE is associated with numerous comorbidities and risk factors, such as glans hypersensitivity, aging, smoking, alcohol consumption, psychological disorders, diabetes, and hypertension.

**Figure 1:**
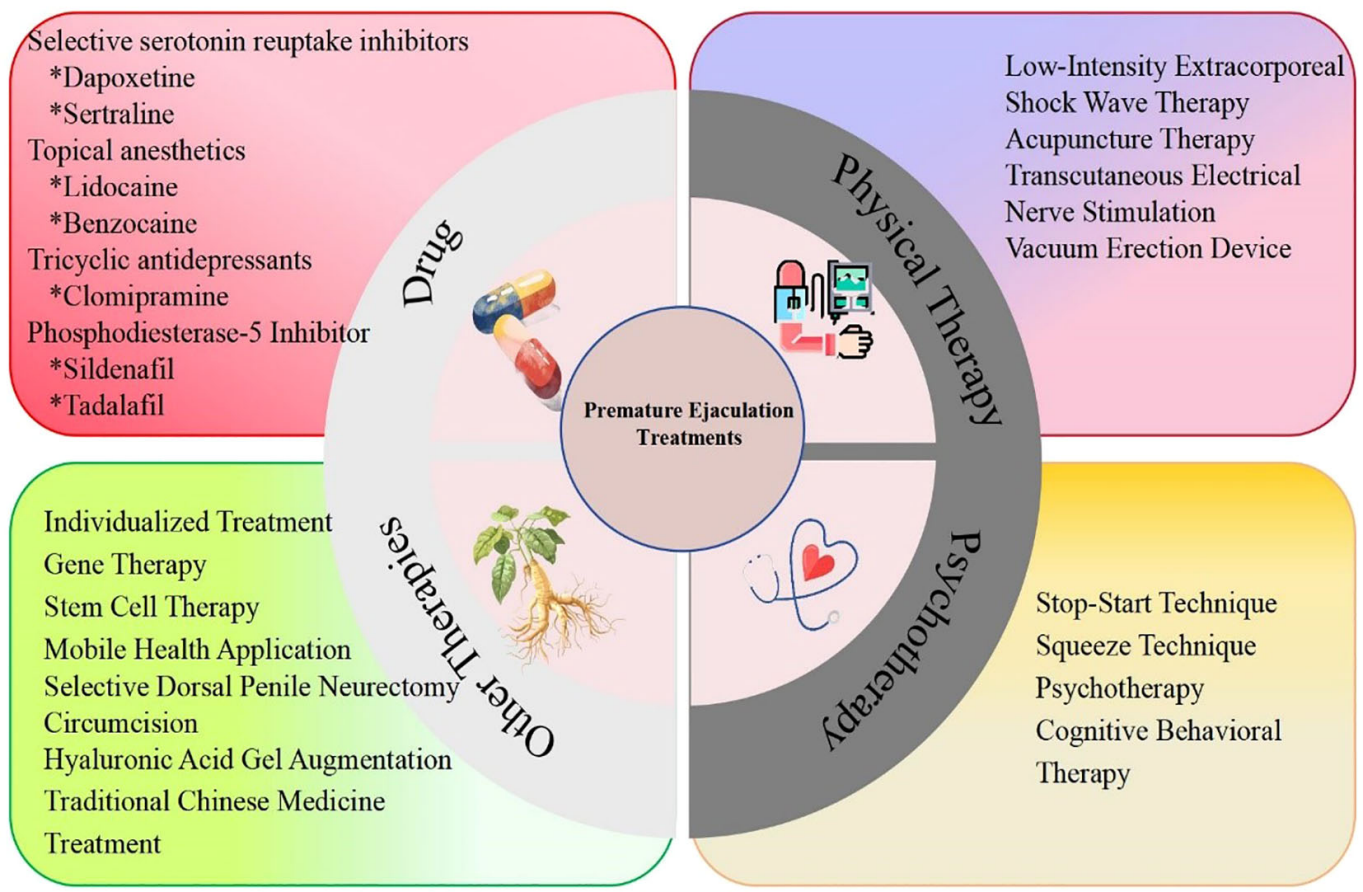
Treatment for premature ejaculation.

The normal ejaculation process is a complex physiological event involving the central nervous system, peripheral nerves, the endocrine system, and the reproductive system (5). The first step in treating PE often involves lifestyle modifications, such as smoking cessation, limiting alcohol intake, exercise, and avoiding staying up late. When PE is primarily caused by emotional or psychological factors, alleviating anxiety and using mood-regulating medications can be effective. Simultaneously, partner involvement is a positive treatment approach. Current therapeutic options for PE mainly include oral selective serotonin reuptake inhibitors (SSRIs), topical anesthetics, psychological therapy, behavioral therapy, device-assisted therapy, traditional Chinese medicine, combination therapy with PDE5 inhibitors, low-intensity shockwave therapy, percutaneous tibial nerve stimulation, and surgical treatment (**Figure 1**). In clinical practice, the most appropriate choice can be made based on the patient’s specific situation. Among these, SSRIs remain one of the most widely used clinical treatments due to their on-demand use, clear efficacy, and rapid onset of action (6). However, some patients experience no response or low responsiveness, along with potential adverse effects such as headache, drowsiness, insomnia, and tremors, which drive the exploration for safer and more targeted treatment methods (7, 8). This article will detail the various treatment methods for PE in conjunction with current research progress.

## Pharmacotherapy: selective serotonin reuptake inhibitors

Selective serotonin reuptake inhibitors (SSRIs) work by selectively inhibiting the reuptake of serotonin (5-HT) into the presynaptic neuron. This increases the concentration of 5-HT in the synaptic cleft, thereby enhancing serotonergic neurotransmission. The prolonged action of 5-HT on postsynaptic receptors inhibits the ejaculatory reflex arc, ultimately delaying ejaculation (9). SSRIs such as paroxetine, fluoxetine, and sertralin have been shown to significantly increase the intravaginal ejaculatory latency time (IELT). In the physiology of ejaculation, the 5-HT-1a receptor plays an activating role, whereas the activation of 5-HT1b and 5-HT2c receptors exerts an inhibitory effect on ejaculation (10, 11). Furthermore, studies have found that daily administration of SSRIs is more effective than on-demand use in delaying ejaculation, supporting the crucial role of 5-HT in modulating the ejaculatory reflex (12). Clinical trials have also confirmed that paroxetine prolongs intercourse duration and improves sexual satisfaction in patients with premature ejaculation (PE), effects which are correlated with the enhancement of serotonergic neurotransmission (13). Therefore, increasing synaptic 5-HT concentration is an effective pharmacological intervention for PE.Clinically used SSRIs can be categorized into on-demand and daily-use types, a distinction closely related to their pharmacokinetic profiles. Dapoxetine, with its short half-life, was the first SSRI specifically developed for on-demand use in PE. In contrast, paroxetine and sertraline have longer half-lives and are suitable for daily administration. Studies indicate that steady-state plasma concentration of paroxetine is achieved approximately 13 days after initiation of daily dosing (14).

SSRIs are the first-line treatment for premature ejaculation, particularly suitable for primary premature ejaculation or secondary premature ejaculation when combined therapy and behavioral therapy prove ineffective (15).SSRIs are first-line pharmacological treatments for PE, particularly when behavioral therapy is ineffective (15). Combination therapy of SSRIs with phosphodiesterase-5 (PDE-5) inhibitors, such as tadalafil, has been shown to further prolong ejaculatory latency compared to SSRIs alone. One clinical study demonstrated that the combination of tadalafil with either paroxetine or dapoxetine was more effective than SSRI monotherapy, especially in younger patients (16). It should be emphasized that dapoxetine is currently the only drug approved for the treatment of premature ejaculation, while other SSRIs are used off-label (17). Compared to dapoxetine, drugs such as paroxetine, sertraline, and fluoxetine were originally developed as antidepressants and are used clinically to achieve delayed ejaculation by leveraging their side effects (18). However, unlike dapoxetine’s rapid absorption and elimination profile, these alternatives exhibit slower onset of action and longer half-lives. Daily dosing is required to achieve stable blood concentrations, potentially leading to more frequent or prolonged side effects such as dizziness, nausea, and delayed ejaculation (19, 20). Furthermore, long-term use may impair sperm quality and induce withdrawal symptoms (21, 22). Although both combination therapy and SSRI monotherapy may cause adverse effects—such as dizziness, nausea, delayed ejaculation, or decreased libido—these are generally tolerable (23). In summary, SSRIs represent a first-line option for PE treatment. They offer significant advantages and can be chosen based on individual differences and needs, either for on-demand or daily use. Future research should continue to explore mechanisms beyond the 5-HT pathway to optimize treatment strategies.

## Tricyclic antidepressants

Tricyclic antidepressants (TCAs) primarily achieve delayed ejaculation by inhibiting the reuptake of serotonin (5-HT) at the presynaptic membrane of neurons, thereby enhancing 5-HT concentration and neurotransmission. Among these, clomipramine is the most widely used and studied representative drug in clinical practice. Choi, JB et al. (24) randomly assigned 159 patients with premature ejaculation to a placebo group (n=53) and a clomipramine group (n=106). After 12 weeks of treatment, the clomipramine group showed significantly improved IELT and PETT scores compared to the placebo group. However, 15.7% of patients experienced nausea and 4.9% reported dizziness. This may relate to clomipramine’s lack of high selectivity for 5-HT reuptake inhibition compared to SSRIs. Its dual reuptake effects on both 5-HT and norepinephrine position it as a second-line treatment when SSRIs prove ineffective (25).

Current evidence indicates clomipramine significantly prolongs ejaculatory latency, demonstrating favorable efficacy in premature ejaculation treatment. However, TCAs are prone to causing anticholinergic side effects and cardiovascular risks, such as dry mouth, constipation, blurred vision, dizziness, and orthostatic hypotension (26, 27). Therefore, developing highly selective dual reuptake inhibitors to reduce anticholinergic and other side effects is essential. Nevertheless, TCAs remain particularly suitable for treating secondary premature ejaculation in patients with comorbid depression and premature ejaculation.

## Phosphodiesterase-5 inhibitors

Phosphodiesterase-5 (PDE-5) inhibitors are classic medications for treating erectile dysfunction. Examples include sildenafil and tadalafil, whose mechanisms of action are likely closely related to inhibiting cGMP degradation, increasing cavernous smooth muscle relaxation, and enhancing arterial blood flow (28). First, PDE-5 inhibitors enhance penile rigidity, effectively boosting sexual confidence and alleviating anxiety, thereby indirectly improving ejaculatory control. Second, these drugs may elevate the ejaculatory threshold by reducing sympathetic nerve tension in the urethra, prostate, and other regions (29).

PDE-5 inhibitors are indicated for secondary premature ejaculation, specifically in patients with concurrent ED. Evidence indicates that in a randomized trial of 160 patients with ED and PE divided into a tadalafil group (n=80) and a placebo group (n=80), after 3 months of daily oral administration of 5 mg tadalafil, the mean (standard deviation) IELT in the tadalafil group increased from 37 (11.24) seconds to 120.5 (47.37) seconds. demonstrating that PDE-5 inhibitors significantly prolong ejaculation time while improving erectile function (30). Furthermore, as demonstrated by Mohseni Rad, H. et al., combination therapy with PDE-5 inhibitors and SSRIs exhibits synergistic effects, proving more effective than monotherapy in terms of IELT. This may be attributed to complementary regulatory actions at both neurovascular and central nervous system levels (16). Consequently, PDE-5 inhibitors offer an additional therapeutic option for treating premature ejaculation, particularly in patients with coexisting erectile dysfunction.

## Topical local anesthetics

Topical local anesthetics are a common approach for treating premature ejaculation (PE). These agents, which include lidocaine, procaine, benzocaine, tetracaine, and various compound formulations, offer advantages such as convenience of use, rapid onset of action, and minimal systemic side effects. They are particularly suitable for managing both primary and secondary PE caused by glans hypersensitivity (31). Studies have indicated that using an anesthetic spray containing lidocaine and prilocaine—applying three sprays five minutes before intercourse—can effectively prolong ejaculatory latency and yield higher sexual satisfaction (32). These agents function by blocking sodium channels in specific areas such as the glans and coronary sulcus, thereby reducing neural impulses and diminishing sensory input from the frenulum and coronal region to achieve local anesthesia (33).

In a randomized controlled trial involving 150 PE patients, the experimental group received a 5% lidocaine spray, while the control group received an alcohol-based placebo spray. Results demonstrated that topical application of 5% lidocaine spray to the glans 10–20 minutes before intercourse improved Acquired Premature Ejaculation (AIPE) scores, intravaginal ejaculatory latency time (IELT), and intercourse frequency compared to the control group. Only tolerable local adverse effects were reported (34).

Although topical local anesthetics are generally considered safe in PE treatment, they are not without adverse effects. The most common issue is excessive numbness of the glans, which may reduce sexual pleasure. A small number of patients may experience local itching or erythema, but these symptoms typically resolve quickly after washing the agent off (35, 36). Benzocaine, another local anesthetic, can reduce penile sensitivity and prolong ejaculatory latency. For patients with high glans sensitivity, using benzocaine-containing condoms or benzocaine wipes may serve as an alternative treatment option (37).

In summary, topical anesthetics can effectively treat both primary and secondary premature ejaculation, particularly for patients with penile glans hypersensitivity or those who are intolerant to oral medications or unwilling to undergo oral drug therapy. In clinical practice, different formulations can be selected based on individual preference, and the dosage can be adjusted according to efficacy and tolerance. These agents effectively reduce penile sensitivity and extend ejaculatory latency. Although side effects such as reduced sexual pleasure may occur, they are generally transient and manageable, underscoring the safety and efficacy of this treatment approach.

## Psychological and behavioral therapies

With the ongoing refinement of healthcare systems and increasing societal life pressures, psychological and behavioral therapies have gradually become core non-pharmacological approaches for managing Premature Ejaculation (PE). This treatment modality aligns well with the modern medical model of “Biopsychosocial” integrated care and is particularly suitable for PE patients experiencing issues such as intercourse-related anxiety, lack of sexual confidence, or other associated psychological concerns (38, 39).

Cognitive behavioral therapy (CBT) has demonstrated positive effects in psychotherapy. Existing evidence indicates that after 8 to 12 CBT sessions for 15 patients, improvements were observed in ejaculatory latency, sense of control, Arab Premature Ejaculation Index, and sexual satisfaction (40). Furthermore, a meta-analysis comparing 653 patients receiving combined CBT and SSRIs therapy with 590 patients using SSRIs alone showed that combination therapy significantly prolonged IELT in PE patients, improved ejaculatory control, scores on the Chinese Index of Premature Ejaculation, and sexual satisfaction, without a significant increase in adverse effects (41). CBT focuses on psychological cognition and emotional regulation. Utilizing individual and partner-assisted counseling, it addresses psychological and behavioral issues related to PE through various techniques, including cognitive restructuring, behavioral skills training, psychoeducation, and exposure therapy (42, 43). This approach helps correct misconceptions, such as equating occasional rapid ejaculation with sexual incompetence or an excessive focus on duration perfection, while guiding partners from a critical to a supportive interaction pattern, thereby avoiding the vicious cycle where sexual performance pressure exacerbates PE.

Behavioral therapy is a commonly used approach for treating PE during sexual activity, aiming to reduce penile sensitivity and excitability of the ejaculatory center through behavioral training. Techniques include the stop-start technique, squeeze technique, penile root masturbation (PRM), pelvic floor muscle training (PFMT), and Kegel exercises (44). The stop-start technique is one of the most frequently used partner-assisted behavioral methods. It involves ceasing stimulation when nearing the ejaculatory threshold during sexual activity, resuming once the excitement subsides, and repeating this process to effectively prolong IELT. The mechanism is related to interrupting stimulation to break the “excitement-ejaculation” reflex (45). Behavioral therapies can significantly prolong IELT, enhance ejaculatory control, alleviate PE symptoms, and also improve partner sexual function and overall sexual quality. Additionally, techniques such as squeezing the glans and coronary sulcus near the ejaculatory threshold to inhibit the ejaculatory reflex, or applying pressure and rubbing at the penile root to prolong pleasure accumulation, can help ameliorate PE symptoms. A study involving 35 PE patients who showed no improvement after 6 months of pharmacotherapy found that after 3 months of PRM training (3 sessions per week), their Premature Ejaculation Diagnostic Tool (PEDT) score decreased from 16.26 to 10.63, and IELT increased from 50 seconds to approximately 192 seconds, indicating that behavioral therapy can alleviate psychological distress and improve ejaculatory control (44).

Psychological and behavioral therapies offer significant advantages, including high safety profiles and broad applicability, making them suitable for subjective premature ejaculation, secondary premature ejaculation, and naturally occurring variations of premature ejaculation. However, challenges include poor patient adherence, low partner cooperation, and minor issues such as unstable pleasure perception or post-emotional release fatigue in a minority of patients during the initial training phase, though these typically require no specific intervention. The methodological limitations primarily include the subjectivity of efficacy assessment; constraints on psychotherapy due to time, financial, and other resource limitations stemming from a shortage of mental health professionals; and the complexity of comorbid conditions such as physiological premature ejaculation or premature ejaculation combined with erectile dysfunction. Additionally, the slow onset of effects may result in suboptimal improvement when relying solely on psychotherapy. This implies that in clinical practice, it may not meet the needs of all patients. Behavioral and psychological therapies serve as the primary treatment for premature ejaculation. For refractory cases, simultaneous intervention at both psychological and physiological levels may be employed. Combining behavioral-psychological therapy with oral SSRIs may improve premature ejaculation (38).

## Physical therapy

Physical therapy for premature ejaculation (PE) primarily encompasses non-invasive techniques, such as low-intensity extracorporeal shockwave therapy (Li-ESWT), acupuncture, and transcutaneous electrical nerve stimulation (TENS). The occurrence of PE is associated with glans hypersensitivity, abnormal local nerve conduction, and impaired microcirculation.

Li-ESWT can improve penile blood supply, promote nerve regeneration and repair, and inhibit hyperactive nerve excitability, making it a non-invasive, safe, and effective treatment method (46, 47). One study involving 212 men with lifelong PE divided participants into four groups; the group receiving combined Li-ESWT and dapoxetine showed significant improvements in the fold increase of intravaginal ejaculatory latency time (IELT), scores on the premature ejaculation-related personal distress scale, and the Clinical Global Impression of Improvement (CGI-I) scale (48). Concurrently, studies indicate that Li-ESWT can enhance the expression of brain-derived neurotrophic factor (BDNF) and nerve growth factor (NGF). By promoting the repair and regeneration of penile nerves and improving microcirculation in the glans, it thereby increases the ejaculatory threshold (49). In summary, Li-ESWT is a promising treatment for PE, effectively regulating local nerve function and raising the ejaculatory threshold. However, current mechanistic research largely focuses on its benefits for erectile dysfunction, and the specific mechanisms of Li-ESWT action in PE are not fully understood, necessitating further exploration through large-sample, multicenter randomized controlled trials.

Acupuncture is recognized by Western clinicians as an alternative therapy for PE, and several studies have evaluated its effectiveness. Acupuncture works by stimulating specific meridian points in the body to regulate the release of neurotransmitters and endocrine hormones (50). A randomized controlled trial involving 90 PE patients, divided into paroxetine, acupuncture, and placebo groups, found that both the paroxetine and acupuncture groups could reduce Premature Ejaculation Diagnostic Tool (PEDT) scores and delay IELT. Although the effect of the acupuncture group was weaker than the paroxetine group, it still demonstrated that acupuncture can delay IELT (51). Another study involving 120 PE patients, randomized into dapoxetine, acupuncture, and sham acupuncture groups, found that while the acupuncture group’s improvement in IELT and PEDT scores was less than the dapoxetine group, it was superior to the sham acupuncture group, indicating that acupuncture has a role in improving these parameters (52). It is important to note, however, that the efficacy of acupuncture for PE remains contentious and requires further investigation.

Transcutaneous electrical nerve stimulation (TENS) is a non-invasive therapy that delivers low-frequency pulsed electrical currents to specific nerve distribution areas, modulating nerve function to alleviate PE symptoms (53). A study showed that when 50 men with lifelong PE were randomly assigned to a transcutaneous posterior tibial nerve stimulation (TPTNS) treatment group or a sham TENS group, the TPTNS group (receiving stimulation three times per week for three weeks) showed improved scores on the Arabic Index of Premature Ejaculation and a median IELT increase of 3.1-fold (54). PE is related to the excessive conduction of sensory signals from the glans and hyperexcitability of the central ejaculatory reflex. By activating Aβ sensory nerve fibers in the penile and lumbosacral skin, TENS can inhibit the transmission of sensory signals to the spinal cord and cerebral cortex, thereby reducing glans sensitivity (55). Additionally, TENS may achieve balance in the ejaculatory reflex by modulating neurotransmitter release (56).

The vacuum erection device (VED) facilitates blood flow into the penile corpora cavernosa, inducing erection and prolonging intercourse duration (57). Although VED is primarily used for treating ED, it may positively impact PE by improving erectile function and boosting self-confidence, thereby enhancing sexual function and psychological state (58). However, no direct studies currently demonstrate that VED can directly improve PE. The use of the device requires active patient cooperation and proficiency, thus adequate guidance and support should be provided in clinical applications.

In summary, for neurosensory primary premature ejaculation with drug intolerance or reluctance to undergo medication, as well as secondary premature ejaculation with localized sensory abnormalities, physical therapy serves as a safe and convenient adjunctive treatment option. However, it also faces challenges such as potential insufficient patient adherence and the need for regular, standardized sessions to maintain effects. Currently, physical therapy can serve as a foundational or alternative treatment for PE, and combination with pharmacological treatments like SSRIs may yield better outcomes.

## Surgical treatment

Surgical treatment for premature ejaculation primarily improves ejaculatory control by adjusting the nerve distribution and sensitivity of the penis. It is suitable for primary premature ejaculation that has not responded to medication or behavioral therapy.Surgical intervention for premature ejaculation (PE) primarily aims to improve ejaculatory control by modulating penile nerve distribution and sensitivity. Surgical approaches include selective dorsal neurectomy, circumcision, and hyaluronic acid gel glans augmentation (59–61).One study involving 314 patients with primary PE who underwent selective dorsal neurectomy reported that the mean number of dorsal penile nerves identified was 7.0 ± 1.9. Postoperatively, significant improvements were observed in intravaginal ejaculatory latency time (IELT) and sexual satisfaction rates compared to preoperative levels (62). This suggests that reducing penile sensitivity by decreasing nerve impulse transmission can effectively prolong ejaculatory latency. Animal models have also demonstrated that dorsal penile neurectomy significantly raises the ejaculatory threshold and improves sexual behavior performance (63).

Furthermore, circumcision may have a certain association with alleviating PE symptoms. By increasing glans exposure and reducing its hypersensitivity, circumcision can potentially extend IELT. A clinical study evaluating 575 circumcised men and 623 uncircumcised controls with PE at 3, 6, 9, and 12 months found that the circumcision group showed significant improvements in IELT, ejaculatory control, and satisfaction, indicating a significant correlation between circumcision and PE development (64). However, a meta-analysis comprising 10,019 circumcised men and 11,570 uncircumcised controls across five subgroups found that circumcision did not significantly improve IELT, suggesting it has no substantial effect on PE (65). This indicates that while circumcision might benefit some PE patients, it is not universally applicable.

Hyaluronic acid injection is a newly developed minimally invasive therapy for PE. It works by “physically thickening the tissue” to buffer stimulation and reduce nerve signal conduction, thereby improving PE symptoms. A clinical study randomizing 80 PE patients into hyaluronic acid and saline injection groups found that at 1, 3, and 6 months follow-up, the hyaluronic acid group showed a significant increase in glans circumference, alongside progressively higher patient and partner satisfaction scores (66). Unlike dorsal neurectomy, the core advantage of hyaluronic acid injection lies in its minimally invasive nature and low trauma. However, further research is necessary to evaluate its long-term efficacy and safety for treating PE patients.

Although surgical interventions provide an alternative for patients with refractory PE, more high-quality evidence-based medical support is required before they can be widely adopted in clinical practice.

## Traditional Chinese medicine treatment

In the realm of Traditional Chinese Medicine (TCM) treatment, topical herbal preparations have garnered increasing attention for managing premature ejaculation. Existing evidence suggests that certain topical TCM formulations, such as herbal sprays (composed of Asarum, Clove, Mantis Ootheca, Aconite, Zhi Shi, Rosehip, Hawthorn Fruit, and Sichuan Pepper Fruit Peel), exhibit kidney-tonifying and essence-consolidating effects. In a clinical controlled study, Ying-Dong Cui (67) randomly assigned 90 patients with premature ejaculation to three groups: Traditional Chinese Medicine group (herbal spray, n=30), desensitization therapy group (automated device, n=30), and combined therapy group (herbal spray + desensitization therapy, n=30), with continuous treatment for 6 weeks. Results showed overall efficacy rates of 65.5%, 67.9%, and 89.7% for the TCM, desensitization therapy, and combined groups, respectively. The combined group demonstrated significantly greater improvements in IELT and the Chinese Premature Ejaculation Index compared to controls, indicating that the herbal spray prolongs ejaculatory latency through local anesthesia and kidney-tonifying effects. The advantages of such treatments lie in their direct local action, sustained efficacy, and relatively minor side effects. However, they require long-term adherence and exhibit slower onset, making them suitable for diverse patient needs. Furthermore, the efficacy and safety of numerous topical Chinese herbal formulations have been supported by clinical studies, positioning them as adjunctive treatment options for premature ejaculation.

Compound TCM formulas also play a significant role in treating premature ejaculation (68). According to TCM theory, premature ejaculation is closely associated with multiple factors including kidney deficiency, blood stasis, phlegm-dampness, and qi stagnation. Consequently, numerous TCM decoctions and acupuncture treatments aim to achieve effects such as tonifying the kidneys and boosting qi, promoting blood circulation and resolving stasis, draining dampness and eliminating phlegm, and harmonizing the viscera (69). Consequently, TCM formulas are a viable choice for primary premature ejaculation stemming from visceral disharmony and secondary premature ejaculation associated with emotional distress or stress. Yu et al. (70) demonstrated that continuous oral administration of Compound Jinji Granules for 4 weeks significantly improved insertion latency (IL), ejaculation frequency (EF), and mating frequency (MF) in rats. Furthermore, decoctions such as Shugan Yidan Fang and Qiaoshao Prescription exert positive effects in treating premature ejaculation by regulating dopamine and serotonin levels. They are widely recognized for improving male sexual function and prolonging ejaculation time (71, 72). Moreover, traditional Chinese medicine formulas possess advantages such as multi-component composition, multi-targeted effects, and adaptability to individual patient symptoms, further enhancing the specificity and efficacy of premature ejaculation treatment. The above findings indicate that integrated Chinese and Western medicine therapy is more effective than SSRIs alone, offering a more comprehensive treatment approach for patients with premature ejaculation (73).

## Future research directions

### The potential of personalized treatment

With social development and in-depth research on premature ejaculation (PE), personalized treatment has gained increasing attention. A growing number of patients present with intertwined physiological, psychological, and social factors (74). Single treatment modalities are no longer sufficient to meet clinical demands. Therefore, future research must establish a multidimensional, comprehensive treatment approach as the core principle, alongside personalized therapeutic strategies tailored to different etiological types and individual variations (75). Developing tailored treatment plans based on patient age, psychological state, and relationship dynamics significantly enhances premature ejaculation management. For patients exhibiting neurotransmitter imbalances (e.g., serotonin, dopamine), phimosis accompanied by sexual confidence deficits, or poor marital sexual quality, treatment should adopt a multidimensional physiological-psychological-social approach: Select SSRIs or similar medications to replenish serotonin and dopamine levels, concurrently performing circumcision to reduce local sensitivity. Psychologically: Enhance mental health counseling to boost patient confidence. Socially: Provide communication skills training for sexual partners and reduce social pressures. Future research will focus on individual patient differences, combining symptom profiles to optimize treatment plans, thereby achieving higher treatment success rates.

### Research on emerging therapies

Gene therapy and stem cell therapy represent emerging approaches in PE treatment with considerable potential (76). Genetic variations may be associated with the occurrence of PE, and gene therapy aims to treat the condition by altering the genetic material of cells, potentially opening new avenues for PE management (77). Stem cell therapy may help repair damaged nerves or tissues through self-renewal and differentiation capabilities, thereby improving symptoms in PE patients (78). Although stem cell therapies have shown promising results in various animal models, most have not met expectations in clinical trials (79). Future research should emphasize the safety, efficacy, and long-term follow-up of these emerging treatments.

### The application of technology in PE management

The rise of mobile health applications offers PE patients tools for self-monitoring, sexual education, and improved doctor-patient communication, enhancing engagement and treatment adherence. One study reviewed nine mobile health applications that provided information on PE diagnosis and treatment, analyzing user participation and functionality (80). These applications showed significant improvements in symptom management and psychological support. Future research should focus on enhancing the scientific rigor and user experience of these apps to ensure accuracy and practical utility.

## Conclusion

In summary, premature ejaculation (PE) is a common male condition that significantly impacts the quality of life of patients and their partners. Currently, oral selective serotonin reuptake inhibitors (SSRIs) are first-line pharmacological treatments for PE, offering high safety and well-established efficacy. For patients intolerant or unresponsive to SSRIs, alternative treatments such as topical anesthetics, behavioral therapy, psychological interventions, surgical options, traditional Chinese medicine, and other emerging therapies may be considered. Although multiple promising treatments are regarded as important future strategies for PE, large-sample, multicenter, high-quality clinical studies are urgently needed to verify their safety and effectiveness. PE is a complex disorder involving psychological, neurological, endocrine, and other risk factors. Treatment should adopt precise and effective personalized strategies based on etiology and individual circumstances.
